# Effects of High CaO Fly Ash and Sulfate Activator as a Finer Binder for Cementless Grouting Material

**DOI:** 10.3390/ma12223664

**Published:** 2019-11-07

**Authors:** Jaehyun Lee, Taegyu Lee

**Affiliations:** Technology Research and Development Institute, Daelim Industrial, Jongno-Gu, Seoul 03152, Korea; archi0528@daum.net

**Keywords:** CaO fly ash, sulfate activator, circulating fluidized bed combustion ash, petro cokes desulfurization gypsum, ground granulated blast-furnace slag, cementless grouting material

## Abstract

The effects or high CaO fly ash and sulfate activator on cementless grouting material were investigated through Labiles Waterglass (LW) grouting applied at an actual construction field. Circulating fluidized bed combustion ash was used as CaO fly ash, and petro cokes desulfurization gypsum was used as sulfate activator. Cementless grouting material (CGM) could decrease the gel time by about 16.7% compared with ordinary Portland cement (OPC). This characteristic improved the average daily workload and construction period per meter by about 13.5% with CGM. Furthermore, when constructing 1000 holes of LW grouting, the construction time could be reduced by 19 days (20% of the total construction period of LW grouting). Meanwhile, CGM could increase the homogel strength by about 48.4% after 28 days compared with OPC. After X-ray diffraction analysis and scanning electron microscope analysis, CGM was found to produce cement hydrate by chemical reaction mechanism of high CaO fly ash and sulfate activator, even though cement was not used. The matrix structure properties of CGM and OPC specimens were similar, but CGM, with 134.3% fineness, exhibited higher compressive strength and lower air permeability than OPC. As a result, CGM could reduce the leakage length per square meter by 74.4% compared with OPC. Using CGM as a substitute for OPC in LW grouting in actual sites could be beneficial in terms of securing construction speed and durability, as well as reducing CO_2_ emissions due to reduction of OPC usage.

## 1. Introduction

The Labiles Waterglass (LW) grouting method is a type of chemical injection method developed to increase water resistance and strengthen grounds by mixing sodium silicate number 3 (SS (No. 3)) solution (liquid A) and ordinary Portland cement (OPC) suspension (liquid B). This method is widely used in Korea, because it enables high strength of consolidation and low permeability at low construction cost [1,2,3].

However, the manufacture of OPC involves a large amount of carbon dioxide (CO_2_) emissions, which is the leading cause of the greenhouse effect in the Earth’s atmosphere [4]. In 2008, CO_2_ emissions in the construction industry were estimated to be about 10% of the total 660 million tons of domestic CO_2_ generated in the reinforced concrete industry [5,6]. Among them, CO_2_ emissions generated by concrete was about 54 million tons, which is about eight times higher than that of rebars [7,8].

To solve the problem of CO_2_ generation, research on geopolymer materials as a substitute for OPC in the concrete industry has been actively conducted [9,10,11]. In particular, the use of admixture materials that can significantly reduce cement clinker production is effective for reducing CO_2_ emissions, because most of the CO_2_ emission generated during the manufacturing process of construction materials are known to occur during cement manufacturing [12,13,14]. However, they are rarely applied in the actual field because of the slow strength development and rapid setting properties, owing to strong alkali [15,16,17].

**Figure 1:**
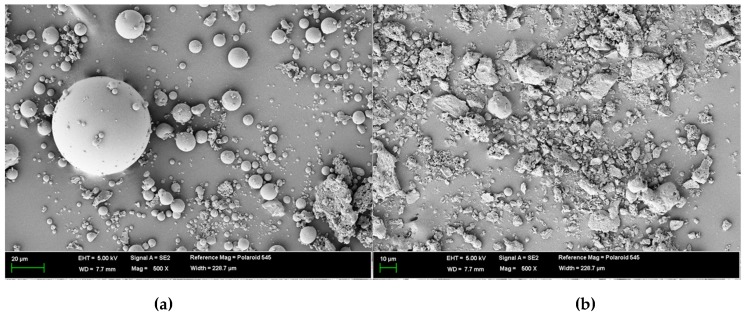
Scanning electron microscope of (**a**) pulverized coal combustion (PCC) ash and (**b**) circulating fluidized bed combustion (CFBC) ash.

Recently, studies on circulating fluidized bed combustion (CFBC) ash have been actively conducted. The chemical composition and characteristics of CFBC ash differ from those of pulverized coal combustion (PCC) ash, which can be attributed to the pulverized coal combustion methods employed in a conventional thermal power plant [18,19,20]. For CFBC ash, the content of free CaO is high and it comprises irregular particles. Thus, it exhibits hydraulic reactivity similar to OPC. On the physical side, as shown in Figure 1, PCC ash is spherical, whereas CFBC ash is irregular and rough [21,22,23,24]. These micrographs of PCC ash and CFBC ash were obtained using the Genesis-2020 scanning electron microscope (Emcrafts, Gwangju-si, Korea).

**Figure 2:**
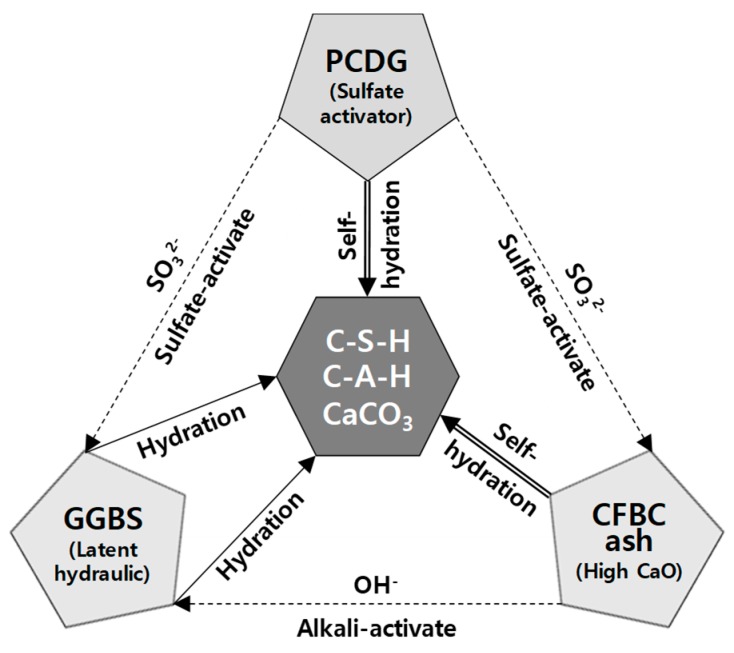
Chemical reaction mechanism of CGM. PCDG, petro cokes desulfurization gypsum; GGBS, ground granulated blast-furnace slag; CFBC, circulating fluidized bed combustion.

On the other hand, in our previous study, we reviewed the engineering properties of cementless grouting material (CGM) that can replace the OPC, an existing binder type of the LW grouting method. There, we reported, the optimal conditions required for CGM, such as the binder type, water/binder (W/B) ratio, and the replacement ratio of Liquid B. CGM is a cement-based grouting inorganic powder (fineness 4190 cm^2^/g) material blended with 50–60% ground granulated blast-furnace slag (GGBS), 30–40% CFBC ash, and 0–20% petro cokes desulfurization gypsum (PCDG) [25]. CFBC ash is a high-calcium fly ash that has a self-hydration property that reacts directly with water and acts as an alkali-activator to stimulate GGBS. PCDG also acts as a sulfate activator, and this chemical reaction mechanism is summarized in Figure 2 [26,27].

However, thus far, no study has attempted to investigate the use of CFBC ash as a substitute for OPC in grouting methods. Furthermore, there have been no papers analyzing the performance and effectiveness of cementless grouting materials using high CaO fly ash and sulfate activator at an actual construction site.

Therefore, this study aims to evaluate the engineering characteristics (gel time, homogel strength, and constructability) and construction management aspects (cost management, schedule management, and quality management) of cementless grouting material (CGM) using CFBC ash by applying it to LW grouting at general construction sites. In this manner, CGM was applied to an actual field, and its contribution to CO_2_ reduction was evaluated.

## 2. Experimental Procedure

### 2.1. Materials

Table 1 lists the physical and chemical properties of SS (No.3) applied as chemical solution of liquid A, comprising H_2_O, SiO_2_, and Na_2_O. The density was 1.384 g/cm^3^, which is slightly heavier than water. The pH of the solution was 14 (strong alkaline property) and its viscosity was 200 cps.

Table 2 lists the physical and chemical properties of OPC and CGM applied as binder liquid B. CGM is an inorganic binder in which GGBS, CFBC ash, and PCDG are mixed in advance. It has high SO_3_ content and fineness as compared to OPC [28].

### 2.2. Procedure

Table 3 summarizes the experimental plan of this study. Liquid binders with a fraction of the mixture were set to two levels, and the rest were set to the same level. Here, the W/B ratio of liquid B was set at 140%, and the volume replacement ratio of liquid B was set at 50%.

The gel state, constructability, and construction speed of the test items were measured to analyze the effects of CGM on shortening gel time. Regarding examining the effects of CGM on increasing homogel strength, hardened state, durability, and leakage length were evaluated.

Table 4 lists the mixture proportions of the LW grouting materials used in the field application. For liquid A, 25 dm^3^ of SS (No.3) and 25 dm^3^ of water were mixed together, and for liquid B, 25 dm^3^ of the binder (OPC, CGM) and 25 dm^3^ of water were mixed together. The injection pressure of LW grouting was 0.3–0.7 MPa, and the materials were injected in 1.5 shots with a double-packer.

Table 5 summarizes the introduction of the project for field application to a new construction site for a knowledge industry center. The underground floors were constructed based on a steel framed reinforced concrete (SRC) structure, and the ground floors based on a reinforced concrete (RC) structure. The land area was approximately 19,486.00 m^2^; construction area, approximately 11,682.09 m^2^; total floor area, 165,153.58 m^2^; and total floor ratio, 445.3%. Furthermore, each building had 938 rooms in 4 basement floors and 10 floors on the ground, and the construction period was 26 months.

**Figure 3:**
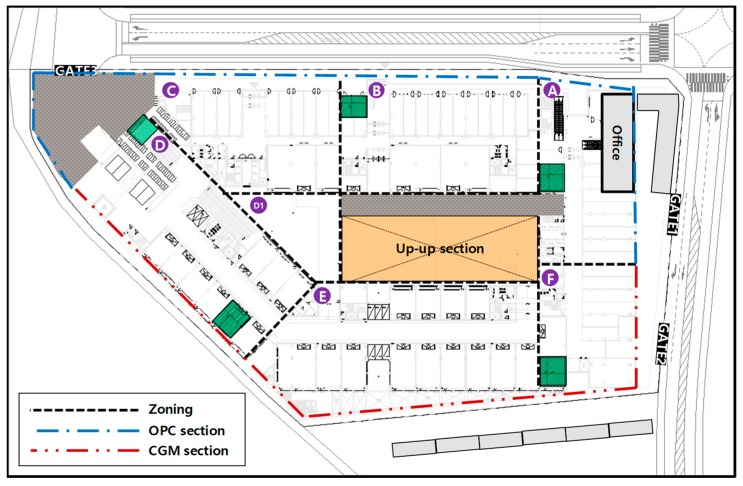
Construction section of OPC and CGM.

Table 6 lists the serial number of H-piles and the sum of cross-sectional area for each OPC and CGM zone. The OPC zone comprises a total of 206 H-piles and a total cross-sectional area of about 6887 m^2^. In addition, the CGM zone comprises a total of 173 H-piles and a total cross-sectional area of about 6321 m^2^. The construction section of OPC and CGM is shown in Figure 3.

### 2.3. Test Methods

#### 2.3.1. Gel Time

The gel time is a major property indicating the characteristics of CGM. When liquids A and B are mixed, the viscosity of the mixture gradually increases, and finally, the fluidity is lost and gelation proceeds. The gel time refers to the time required from the mixing of CGM to the loss of fluidity and start of gelation [29].

The gel time can be measured using two methods: Cup mixing and viscometer methods. In this study, the viscometer method was used for indoor tests, and the cup mixing method was used for the field test [30].

The cup mixing method is described as follows. First, 50 mL of liquids A and B were measured in a beaker or paper cup. Thereafter, the entire amount of liquid B was mixed with liquid A at intervals of about 1 s, and the entire liquid A was mixed with liquid B.

When liquid A and liquid B were continuously stirred, the fluidity was rapidly lost. The time at which the flow of the mixed solution stops was recorded as the gel time, which was measured three times for each type of mixture proportion, and the average value (rounded off at the first decimal place) was calculated.

In addition, the target of gel time was set at 20–50 s by referring to the LW grouting method special specification of Jeong-sun engineering company [31].

#### 2.3.2. Homogel Strength

**Figure 4:**
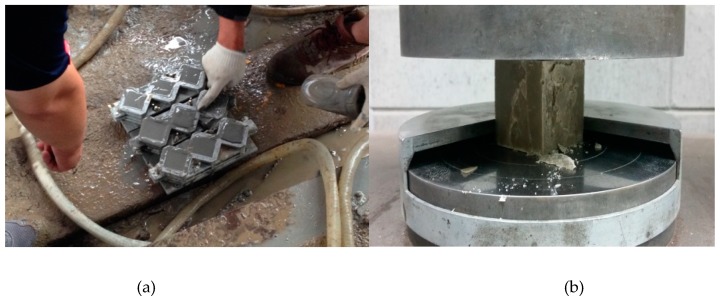
Test methods of homogel strength: (**a**) Molding of cubic molds for homogel strength test; (**b**) homogel strength test.

Figure 4 shows the methods of the homogel strength test. Cubic specimens of 50 mm × 50 mm × 50 mm were produced according to ASTM C109/C109M: 16a (Standard Test Method for Compressive Strength of Hydraulic Cement Mortars) [32]. Specimens were cured in the mold for 24 h; after re-molding, they were cured in a water tank at a curing temperature of 20 ± 2 °C, as per the ASTM C109/C109M. After the 7th day of aging, the mean values of three specimens were calculated for each mixing factor.

The homogel strength is the main property of the grouting material, indicating the compressive strength of the cementitious material obtained by curing solely the mixture of liquids A and B. Therefore, the homogel strength was evaluated by the compressive strength at the initial age of 7 days to determine the progress of the cementation of the grouting material. Generally, the goal is to achieve a homogel strength of at least 2 MPa at 7 days in the construction site.

#### 2.3.3. Chemical Shrinkage and Air Permeability

**Figure 5:**
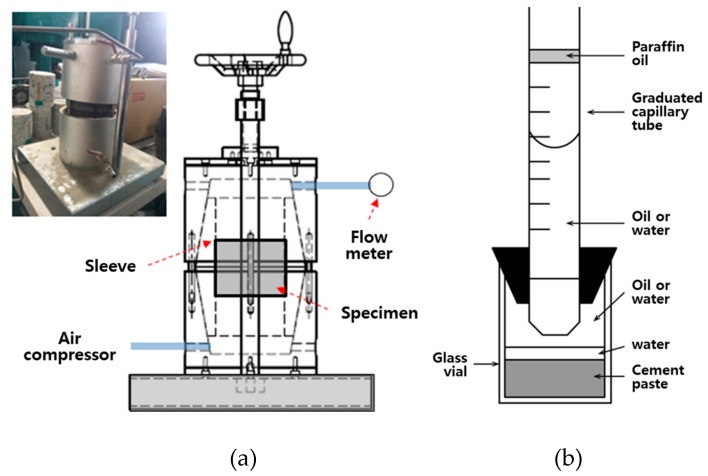
Test methods of (**a**) the air permeability test and (**b**) the chemical shrinkage test.

Figure 5a shows the indoor air permeability coefficient test method used in this study. To determine the air permeability coefficients of OPC and CGM, specimens were prepared by cutting cylindrical shapes in a size of ø100 mm × 200 mm to 50 mm from the center of the compounds at the age of 2 and 4 weeks. These specimens were tested in accordance with KS L 3317, Testing Method for Permeability to Gases of Refractory Products [33]. The air permeability coefficient of specimens was calculated based on Equation (1), and the air permeability coefficient was determined as the average of three specimens. Also, Figure 5b shows the results of the chemical shrinkage test by age, conducted in accordance with ASTM C 1608 [34].

(1) $$\mu =c\times \eta \times \frac{h}{3.14{\left(\frac{d}{2}\right)}^{2}}\times \frac{1}{p_{1}}\times \left(\frac{2\left(p_{0}+p_{1}\right)}{2p_{0}+p_{1}}\right)\times q_{v}$$

where, μ: air permeability coefficient of specimen (m^2^)

*c*: correction factor (1/60 × 10^−6^)
*η*: viscosity of measured gas (Pa·s)
*h*: height of specimen (mm)
*d*: diameter of specimen (mm)
*p*_0_: atmospheric pressure (kPa)
*p*_1_: gas pressure (kPa)
*q_v_*: gas flow rate (cm^3^/min).

#### 2.3.4. Other Test Items

To evaluate the constructability and construction speed, the average daily workload and construction period per meter of the OPC and CGM sections was calculated. For examining durability, X-ray diffraction analysis and scanning electron microscope analysis were performed. Further, leakage length per square meter were measured to assess the leakage length. The protocol for the assessment of the leakage was conducted as follows: Visual observation and recording of the leakage length after rain, followed by calculation of the total construction area of LW grouting wall and calculation of the leakage length per square meter.

## 3. Results and Discussion

### 3.1. Effects of CGM on Shortening Gel Time

**Figure 6:**
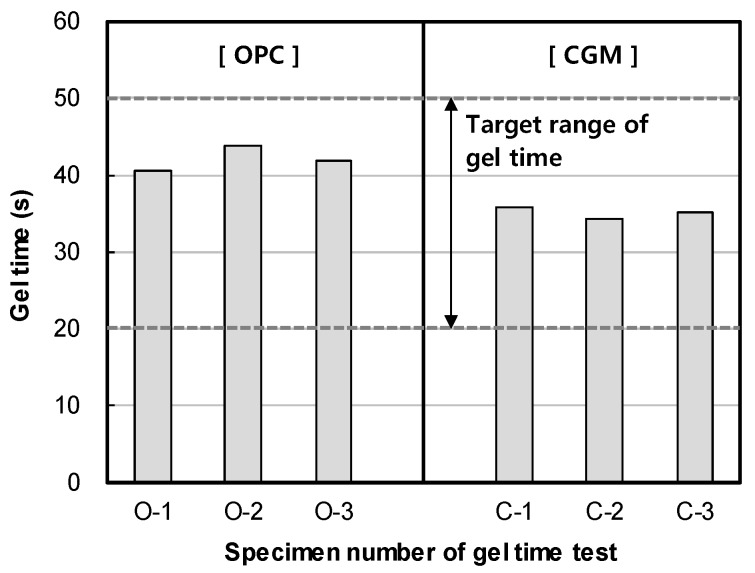
Measurement results of gel time of OPC and CGM.

**Figure 7:**
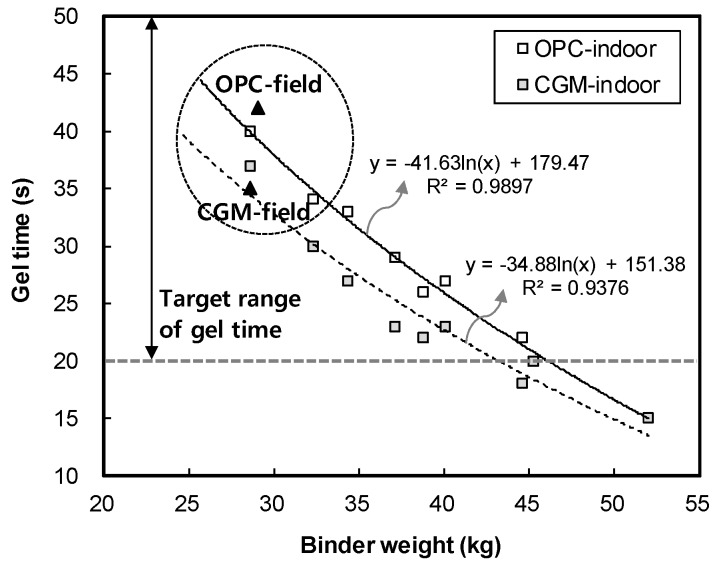
Comparison of field and indoor test results of gel time according to binder weight.

Figure 6 shows the measurement results of gel time for binder type of liquid B. The gel time average of OPC was found to be 42 s, whereas that of CGM was 35 s. Both OPC and CGM satisfied the target range of gel time (20~50 s). Thus, under the same mixture proportions and field conditions, CGM shortened the gel time by 7 s after 7 days, compared with OPC. This is because the fineness of CGM (4190 cm^2^/g) is 134.3% higher than that of OPC (3120 cm^2^/g), and the reaction time with SS (No.3) is accelerated and gel time is shortened. Figure 7 shows the comparison of field (this paper) and indoor [25] test results of gel time according to binder weight, with similar results.

With the application of CGM to the construction site, the grouting step injection could be sped up by shortening the gel time. Table 7 summarizes the results of measuring the average daily workload by binder type liquid B. Using OPC, 95 days were required for constructing a total length of 13,913 m, and the average daily workload was measured as 146.5 m/day. With CGM, 76 days were required for constructing a total length of 12,642 m, and the average daily workload was measured as 166.3 m/day. Thus, the average daily workload of CGM was higher by 19.8 m/day than that of OPC. The measurement of average daily workload was based on 8 h/day (one machine, two workers) of working time.

**Figure 8:**
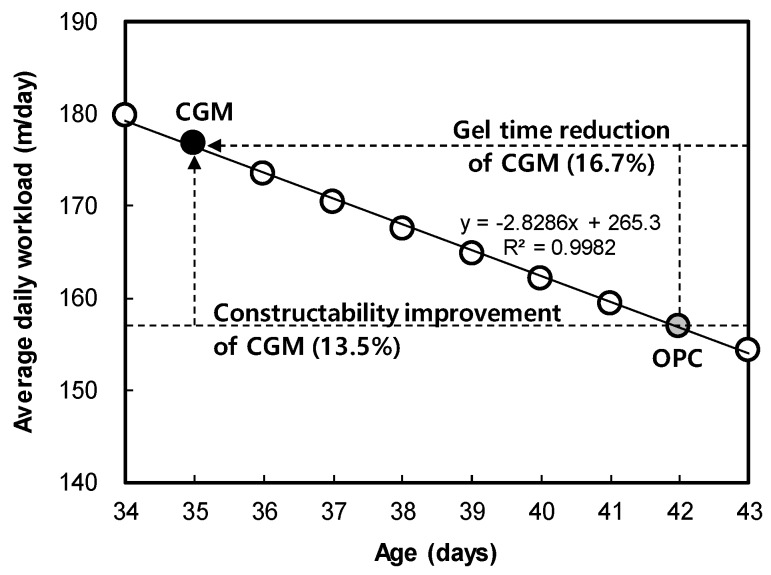
Correlation between gel time and average daily workload.

The main factor affecting the average daily workload was gel time, under the same conditions of OPC and CGM in the field. This is because the grouting step injection rate becomes faster as the gel time is increased, as described above. Figure 8 shows the relationship between gel time and workload, which was derived with the function formula y = −2.8286x + 265.3. CGM reduced the gel time by about 16.7% after 7 days, and the constructability was improved by about 13.5%.

Meanwhile, Table 8 summarizes the analyzed results of the construction period per meter by binder type of liquid B. For OPC, a total construction period of 45,600 min was required for the total length 13,913 m, and the construction period per meter was calculated as 3.28 min. For CGM, a total construction period of 36,480 min was required for the total length 12,642 m, and the construction period per meter was calculated as 2.89 min.

Thus, the construction period of CGM per meter is shorter by 0.38 min compared with OPC. The construction period per meter was calculated based on 8 h/day (one machine, two workers) of working time.

**Figure 9:**
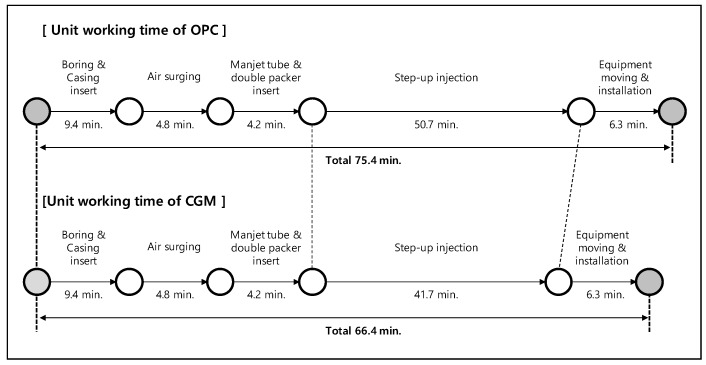
Unit work speed analysis results of OPC and CGM.

To analyze the construction speeds of OPC and CGM, this study analyzed the construction procedure in terms of unit work and measured the average work time per unit work. The results are shown in Figure 9. The injection speed of CGM, which has high fineness, was evaluated to be faster than that of OPC, and the construction speed for one hole (23 m depth) was faster by 113.5%. Thus, when constructing 1000 holes, CGM can shorten the construction time by approximately 19 days compared with OPC (based on 8 h/day).

The results of analyzing the effects of CGM on shortening gel time are as follows: CGM has been shown to reduce the gel time, compared to OPC, by about 16.7%. In the actual field, it was analyzed that constructability (average daily workload) and construction speed (construction period per meter) improved by about 13.5% when applying CGM to LW grouting. As a result, when constructing 1000 holes of LW grouting, the construction time could be reduced by 19 days (20% of the total construction period of LW grouting).

### 3.2. Effects of CGM on Increasing Homogel Strength

**Figure 10:**
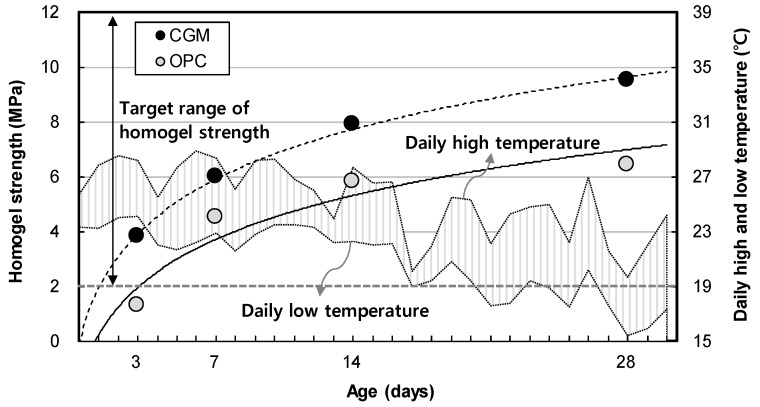
Measurement results of homogel strength of OPC and CGM.

**Figure 11:**
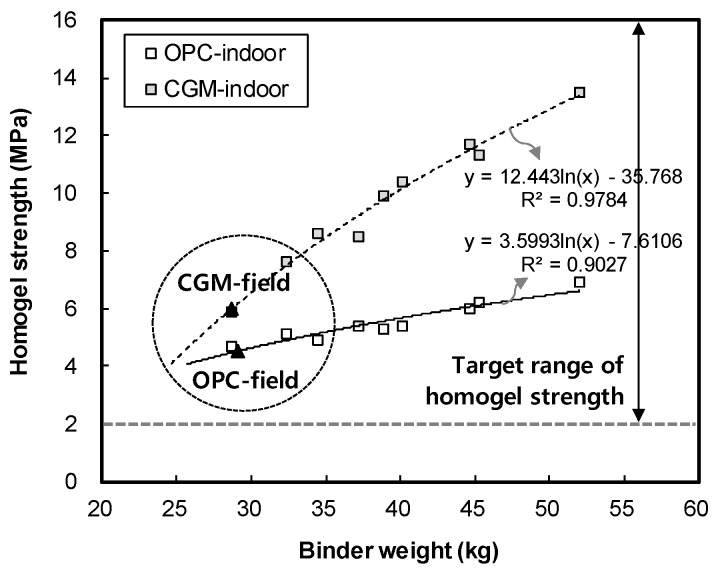
Comparison of field and indoor test results of homogel strength according to binder weight.

Figure 10 shows the measurement results of homogel strength for binder type liquid B. The gel time and homogel strength of OPC was found to be 4.5 MPa, whereas that of CGM was 6.0 MPa after 7 days. Both OPC and CGM satisfied the target range of homogel strength (2.0 MPa or more at 7 days). Thus, under the same mixture proportions and field conditions, CGM increased the homogel strength by 3.1 MPa (48.4%) after 28 days, compared with OPC. Figure 11 shows the comparison of field (this paper) and indoor [25] test results of homogel strength according to binder weight, with similar results to gel time in Section 3.1.

With the application of CGM to the construction site, the homogel strength could be increased to improve the durability of impermeable walls, and the ability to withstand the influence of groundwater through initial strength could be improved.

**Figure 12:**
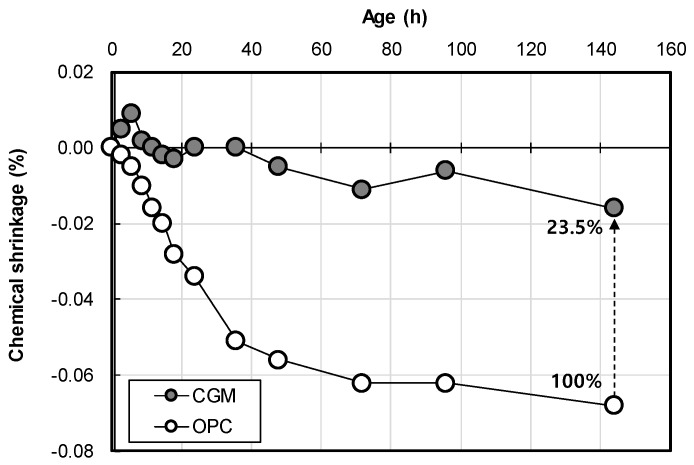
Chemical shrinkage test result of OPC and CGM according to age.

Figure 12 shows the chemical shrinkage test result of OPC and CGM according to age. As a result, the chemical shrinkage of OPC was approximately 0.068 ml/g at 6 days, whereas that of CGM was approximately 0.016 mL/g at the same age. Thus, the chemical shrinkage of CGM was smaller by approximately 23.5% compared to that of OPC. Therefore, as the chemical shrinkage of CGM is 23.5% smaller than that of OPC, it is expected to reduce the possibility of leakage compared to OPC when CGM is applied to the actual field impermeable wall.

**Figure 13:**
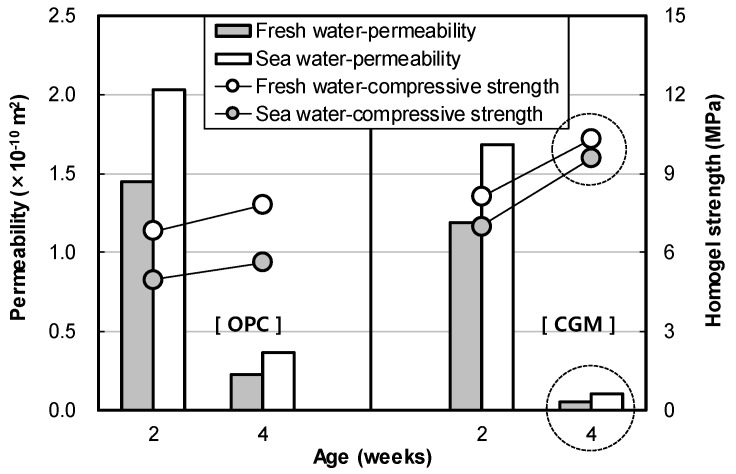
Indoor air permeability coefficient test result of OPC and CGM according to curing water types.

**Figure 14:**
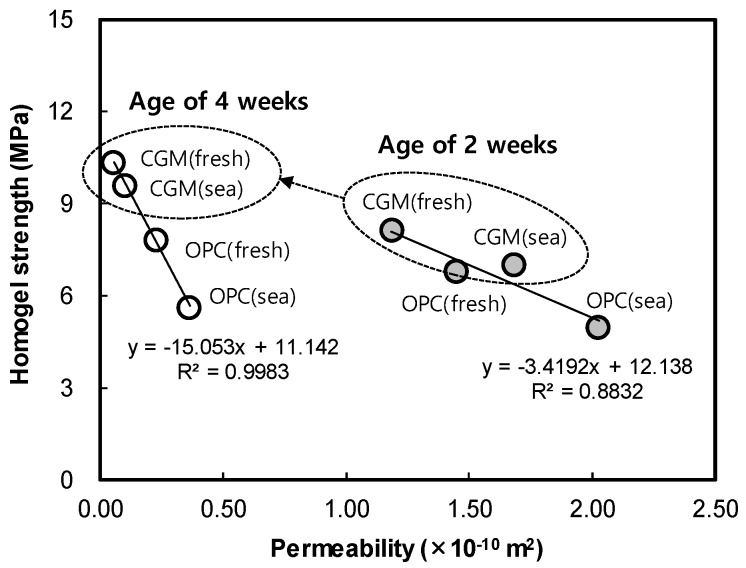
Correlation between permeability and compressive strength according to age.

Figure 13 shows the indoor air permeability coefficient test result of OPC and CGM according to curing water types. In fresh water curing, air permeability of CGM was 81.9% and 23.4% at 2 and 4 weeks of age, and 83.1% and 28.2% at 2 and 4 weeks of sea water curing conditions, respectively. Therefore, CGM was judged to have excellent water tightness under the same curing conditions and excellent performance under sea water curing conditions compared to OPC. Also, Figure 14 shows the correlation between permeability and compressive strength according to age. As permeability decreased by 0.1 × 10^−10^ m^2^, homogel strength increased 0.34 MPa and 1.5 MPa at 2 and 4 weeks of age, respectively. The trend of this graph justifies the principle that matrix structure becomes dense as the homogel strength increases. It was found that the larger the age, the greater the effect of pore amount on the homogel strength, because the air permeability coefficient tends to decrease as the age increases. In particular, homogel strength at 4 weeks of CGM was able to obtain 93% of fresh water curing conditions, even under sea water curing conditions. Therefore, in order to secure durability in the ground, it would be advantageous to apply CGM to OPC.

**Figure 15:**
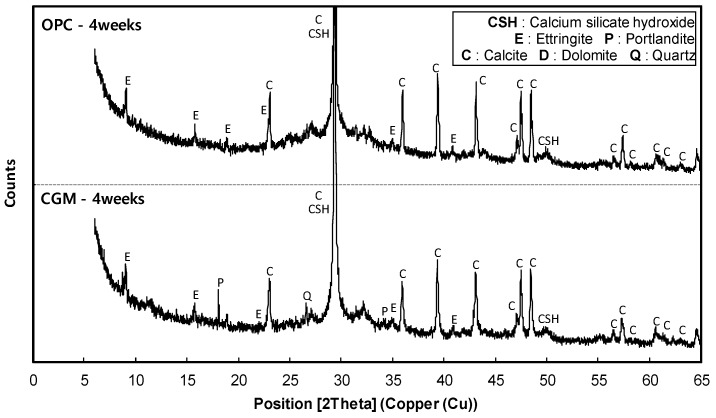
X-ray diffraction analysis results of OPC and CGM at 4 weeks of age.

Figure 15 shows the X-ray diffraction analysis results of OPC and CGM at 4 weeks of age. In the OPC specimen, calcium silicate hydrate (C–S–H), ettringite (Ca_6_Al_2_(SO_4_)_3_(OH)_12_·26H_2_O), and calcite (CaCO_3_) were detected, which are representative hydrates of cement at the age of 4 weeks. On the other hand, in the CGM specimen, portlandite (Ca(OH)_2_) and quartz (SiO_2_) were detected, in addition to C–S–H, ettringite, and calcite. The fact that not only OPC, but also CGM generated C–S–H and ettringite, which are cement hydrates, confirms that CGM generated cement hydrates by hydration, even with no cement. This is also seen in the X-ray diffraction analysis of OPC and CGM, where no detectable differences are observed. The CGM specimen generated portlandite, in addition to the OPC hydrates, after aging of 4 weeks.

**Figure 16:**
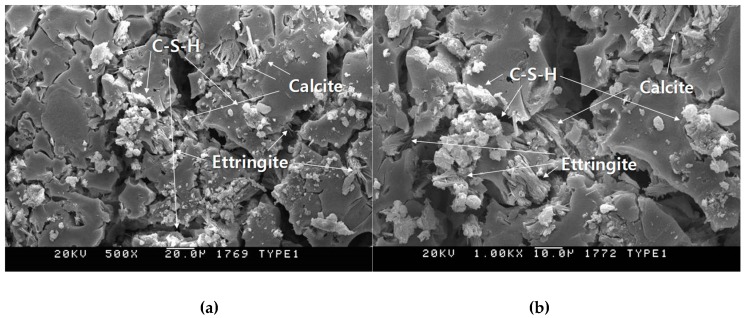

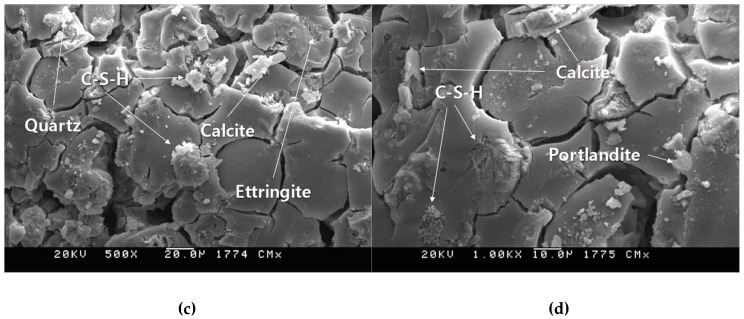
Scanning electron microscope analysis results of (**a**) OPC-500×, (**b**) OPC-1000×, (**c**) CGM-500×, and (**d**) CGM-1000× at 4 weeks of age.

Figure 16 shows the scanning electron microscope analysis results of OPC and CGM aged 2 and 4 weeks. As shown in Figure 15, CGM specimens showed more portlandite (Ca(OH)_2_) and quartz (SiO_2_) than OPC. Since both CGM and OPC are composed of calcium silicate hydrate (C–S–H), ettringite (Ca_6_Al_2_(SO_4_)_3_(OH)_12_·26H_2_O), and calcite (CaCO_3_), the matrix structure properties of CGM and OPC specimens were almost similar. In addition, the overall number and size of cracks were found to be almost similar. This confirmed that a hardened OPC-like matrix structure could be made using the high CaO fly ash and sulphate activator without using cement.

Table 9 summarizes the leakage length measurement results by binder type liquid B. For OPC, the total leakage length for a total area of 6887 m^2^ was 85 m. Thus, the leakage length per square meter was calculated as 0.012 m. For CGM, the total leakage length for a total area 6321 m^2^ was 20 m. Thus, the leakage length per square meter was calculated as 0.003 m. Therefore, the leakage length of CGM per m^2^ was shorter by 0.009 m/m^2^ compared with OPC.

**Figure 17:**
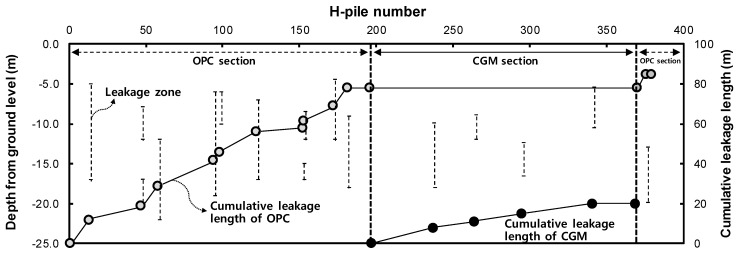
Leakage zone and cumulative leakage length of OPC and CGM according to the number of H-piles.

Figure 17 shows the leakage zone and cumulative leakage length of OPC and CGM according to the number of H-piles. The slope of cumulative leakage length of CGM was smaller than that of OPC. In addition, the total cumulative leakage length of CGM was about 65 m shorter than that of OPC. Figure 7 shows that the gel time of CGM was faster than that of OPC; therefore, CGM was less affected by water in the ground. Moreover, the initial compressive strength of CGM was high, which is advantageous for ensuring the durability of impermeable walls.

The results of analyzing the effects of CGM on increasing homogel strength are as follows: CGM has been shown to increase the homogel strength compared to OPC by about 48.4% after 28 days. From X-ray diffraction and scanning electron microscopy analysis, the matrix structure properties of CGM and OPC specimens were observed to be quite similar. This confirmed that a hardened OPC-like matrix structure could be made using the high CaO fly ash and sulphate activator without using cement. On the other hand, CGM showed a 48.4% increase in compressive strength compared to OPC at 28 days of age. In addition, the air permeability was 23.4% and 28.2% compared to OPC in fresh and sea water curing conditions, respectively. The reason for this seems to be that at the early age, the fineness of CGM is higher than that of OPC, implying that there are more particles within the same volume, and the structure becomes denser by the early reaction of free CaO whose quantity is larger in CGM. As a result, CGM could reduce the leakage length per square meter by 74.4% compared with OPC.

## 4. Conclusions

In this study, the effects or high CaO fly ash and sulfate activator on cementless grouting material were investigated through LW grouting applied at an actual construction field. The following conclusions can be drawn.

(1) CGM could decrease the gel time by about 16.7% compared with OPC. When CGM is applied as a binder for LW grouting in the field, the step injection speed increases, so the average daily workload and construction period per meter increase about 13.5%. As a result, when constructing 1000 holes of LW grouting, the construction time could be reduced by 19 days (20% of the total construction period of LW grouting).

(2) In addition, CGM could increase the homogel strength by about 48.4% after 28 days compared with OPC. The chemical shrinkage of CGM was smaller by approximately 23.5% at 6 days of age compared to that of OPC. Also, the air permeability of CGM was 23.4% and 28.2% of OPC at 4 weeks of age under fresh water curing and sea water curing, respectively. Therefore, CGM showed less pore structure and watertight characteristics than OPC.

(3) After X-ray diffraction analysis and scanning electron microscope analysis, CGM was found to produce cement hydrate by the chemical reaction mechanism of high CaO fly ash and sulfate activator, even though cement was not used. The matrix structure properties of CGM and OPC specimens were quite similar. However, the CGM fineness was 134.3% higher than that of OPC, indicating that the CGM had high compressive strength and low air permeability.

Using CGM as a substitute for OPC in LW grouting in actual sites could be beneficial in terms of securing construction speed and durability, as well as reducing CO_2_ emissions by reducing OPC usage. Our future study will focus on understanding the reactivity properties of CGM and OPC according to chemical and mineralogical composition, the role of pH, the activator, etc. Moreover, we will approach the environmental management aspect of the application of the LW grouting method to examine the environment-friendly characteristics of CGM for CO_2_ reduction and energy conservation.

## Figures and Tables

**Table 1:** Chemical composition and physical properties of chemical liquids used.

Materials	Chemical Composition (%)					Density (g/cm^3^)	pH (at 25 °C)	Viscosity (at 25 °C, Pa·s)
	H_2_O	SiO_2_	Na_2_O	Fe_2_O_3_	WI^(1)^			
SS (No. 3)	63.6	27.2	9.14	0.0034	0.0026	1.384	14	0.2

^(1)^ WI: Water insolubility.

**Table 2:** Chemical composition and physical properties of binders used. OPC, ordinary Portland cement; CGM, cementless grouting material.

Materials	Chemical Composition (%)							Density (g/cm^3^)	Fineness (cm^2^/g)
	SiO_2_	Al_2_O_3_	Fe_2_O_3_	CaO	MgO	SO_3_	Other		
OPC	17.20	4.38	3.13	66.70	3.03	3.48	2.08	3.15	3,120
CGM	17.60	7.01	0.52	58.85	2.02	12.73	1.27	2.89	4,190

**Table 3:** Experimental plan. W/B, water/binder.

Evaluation Items			Experimental Variables
Factors of mixture	Types of liquid chemical		SS (No. 3)
	Types of liquid B binders		OPC, CGM
	W/B ratio of liquid B		140%
	Volume replacement ratio of liquid B		50%
Test items	Effects of CGM on shortening gel time	Gel state	Gel time (s)
		Constructability	Average daily workload (m/day)
		Construction speed	Construction period per 1 m (min/m)
	Effects of CGM on increasing on homogel strength	Hardened state	Homogel strength (MPa)
		Durability	Chemical shrinkage (%)
			Air permeability (×10^−10^ m^2^)
			X-ray diffraction analysis
			Scanning electron microscope analysis
		Leakage length	Leakage length per 1 m^2^ (m/m^2^)

**Table 4:** Mixture proportions of Labiles Waterglass (LW) grouting materials.

Mix No.	Liquid A		Liquid B	
	SS (No.3) (dm^3^)	Water (dm^3^)	Binders (dm^3^)	Water (dm^3^)
OPC	25	25	10	40
CGM	25	25	10	40

Remarks: Injection pressure: 0.3–0.7 MPa; injection method: 1.5 Shot, double-packer injection.

**Table 5:** Introduction of project for field application.

Items	Contents
Project name	Knowledge industry center new construction
Structure system	Under ground floor: steel framed reinforced concrete (SRC) structure; ground floor: reinforced concrete (RC) structure
Land area	19,486.00 m^2^ (5894.52 pyeong)
Construction area	11,682.09 m^2^ (3533.83 pyeong)
Total floor area	165,153.58 m^2^ (49,958.96 pyeong)
Total floor ratio	445.30%
Building scale	4 basement floors–10 floors/1 building/total of 938 rooms
Work period	26 months

**Table 6:** LW grouting sections by binder types.

Binder Types	Serial Number of H-Pile	Sum of Cross Section Area
OPC	1–196, 370–379	6887 m^2^
CGM	197–369	6321 m^2^

**Table 7:** Results of measuring the average daily workload.

Items	Total Length(m)	Work Days (Days)	Average Daily Workload (m/Day)	Remarks
OPC	13,913	95	146.5	Measured based on 8 h/day (one machine, two workers)
CGM	12,642	76	166.3	

**Table 8:** Results of measuring construction period per meter.

Items	Total Length (m)	Construction Period (min)	Construction Period Per Meter (min/m)	Remarks
OPC	13,913	45,600	3.28	Measured based on 8 h/day (one machine, two workers)
CGM	12,642	36,480	2.89	

**Table 9:** Results of measuring leakage length per square meter.

Items	Area (m^2^)	Leakage Length (m)	Leakage Length Per 1 m^2^ (m/m^2^)	Remarks
OPC	6887	85	0.012	Measured based on the length of CIP leak section
CGM	6321	20	0.003	
